# “*Trauma to recovery*”: the role of participatory arts in facilitating recovery from COVID-19-related trauma in care homes

**DOI:** 10.3389/fpsyg.2026.1844988

**Published:** 2026-07-02

**Authors:** Ceri Wilson, Anna Dadswell, Hilary Bungay

**Affiliations:** 1 Faculty of Health, Medicine and Social Care, Anglia Ruskin University, Chelmsford, United Kingdom; 2 Faculty of Health, Medicine and Social Care, Anglia Ruskin University, Cambridge, United Kingdom

**Keywords:** care homes, older people, participatory arts, trauma-informed, wellbeing

## Abstract

**Introduction:**

The Artists’ Residencies in Care Homes (ARCH) programme led by Magic Me involved four arts organisations delivering participatory arts in four UK care homes for older people. The COVID-19 pandemic disrupted the programme, causing substantial trauma to care home communities. Artists paid greater attention to supporting staff and resident wellbeing and recovery from trauma. We present a trauma-informed framework for participatory arts practice in residential care homes, based on learning from the responsive approach of ARCH.

**Methods:**

The four-year programme comprised research and development focused on building relationships and testing ideas, followed by artists’ residencies including workshops, performances, and training in homes, outdoor spaces, and online (e.g., Zoom, film). Artistic media included dance/movement, textiles, music, performance, film, virtual reality, and photography. The research explored how arts and care home staff collaborated to deliver and embed participatory arts in care homes. Qualitative data were collected throughout, comprising observations of meetings, training, and arts activities, alongside interviews and focus groups with artists and care home staff reflecting on their expectations, training/support needs, experiences, and learning. 11 artists, 2 Magic Me directors and 27 care home staff (including home managers, lifestyle coordinators, carers, and representatives from the care home management company) participated. Data was analysed using inductive reflexive thematic analysis.

**Results:**

One theme captures the context and experiences of COVID-19-related trauma in care homes. Subsequent themes describe artist approaches to supporting trauma recovery in care homes: creating safe and inclusive spaces, training and support for artists/facilitators, building trusting and collaborative relationships, empowering care home staff and residents, fostering joy, and channelling the power of the participatory arts. These themes informed a trauma-informed framework for participatory arts practice in care homes.

**Discussion:**

We have proposed a trauma-informed framework for participatory arts in residential care homes, which is of particular importance considering the COVID-19-related trauma that care home communities have faced. As care homes continue to recover from the emotional impacts of the pandemic, and face continuing virus outbreaks and staffing challenges, the participatory arts provide the opportunity to restore joy and promote wellbeing for care home staff and residents.

## 1 Introduction

COVID-19 had a devastating impact on residential care homes globally. In England and Wales, 45,632 COVID-19-related care home resident deaths occurred between March 2020 and January 2022 (Office for National Statistics, 2022). Across European countries, 42–57% of all COVID-19-related deaths were people living in nursing homes, and this was as high as 82% in several US states and Canada (Comas-Herrera et al., 2020). However, these figures do not account for the many residents who were not tested for COVID-19, nor the care home staff who lost their lives. The devastating losses in care homes reflect the “*slow, late and inadequate*” (Daly, 2020, p. 985) response of governments to provide personal and protective equipment (PPE), COVID-19 testing, and clear policy and guidance to care homes at the start of the pandemic (Cremers and Janssen, 2023; McGilton et al., 2020). This left many care home residents and staff feeling abandoned, forgotten, and devalued (McGilton et al., 2020; Cockshott et al., 2024).

During the pandemic, care home staff faced increased workloads, substantial disruption to working practices, and significantly increased responsibilities for end-of-life care and exposure to more deaths, causing emotional trauma with little additional emotional support or time to grieve (Nyashanu et al., 2020; Spacey et al., 2021). Many experienced guilt and moral distress after losing residents they had cared for over many years, considering their own role in COVID-19 transmission and feeling that they had been unable to give high standards of care (Greenberg et al., 2020; McGilton et al., 2020; Cockshott et al., 2024). As a result, there were high levels of anxiety, depression, and post-traumatic stress disorder amongst care home staff (Greene et al., 2021; Beattie et al., 2023), alongside increased levels of depression, anxiety, and loneliness amongst residents (Ekoh, 2021).

Six months before the first UK COVID-19 lockdown, the Artists Residencies in Care Homes (ARCH) programme commenced in Essex, England, led by arts organisation Magic Me with research conducted by the authors from Anglia Ruskin University (ARU). Four arts organisations were paired with four residential care homes for older people, to deliver innovative participatory arts experiences for residents and staff. ARCH comprised a research and development (R&D) phase focused on building relationships and testing ideas, followed by larger-scale artists’ residencies, and a learning and legacy phase. However, as the R&D phase ended, the UK entered the first of three lockdowns. As restrictions began to ease and ARCH restarted, it became clear that the care homes had faced substantial trauma. While the focus remained on delivering high quality arts practice, greater attention was paid to supporting care home staff and resident wellbeing and their recovery from trauma. Wellbeing is understood here as a positive state encompassing both quality of life and being able to “to contribute to the world in accordance with a sense of meaning and purpose” which is determined by social, economic and environmental conditions (WHO, 2021, p. 10).

Trauma results from an event, or events, that is experienced as physically or emotionally harmful or life-threatening and has lasting adverse effects on functioning and mental, physical, social, emotional, or spiritual well-being (Substance Abuse and Mental Health Services Administration [SAMHSA], 2014). It overwhelms an individual’s coping resources, leaving them feeling frightened, vulnerable, and helpless (Newland et al., 2022). A trauma-informed approach to care is not a specific treatment intervention but a way of working which seeks to remove the fear, lack of choice and loss of control caused by trauma, by creating inclusive, safe services that provide opportunities to rebuild a sense of empowerment and control. The most cited trauma-informed framework (Substance Abuse and Mental Health Services Administration [SAMHSA], 2014) is guided by six principles: (1) ensuring settings and interactions promote a sense of safety; (2) building and maintaining trusting relationships; (3) peer support; (4) collaboration and mutuality; (5) empowerment, voice and choice; and (6) cultural sensitivity and inclusive practice. Whilst trauma-informed approaches are more widely adopted in the US, recently they have had growing endorsements in UK policy (Emsley et al., 2022). In 2021 Fenney presented trauma-informed care as a solution to addressing the collective trauma of the COVID-19 pandemic for healthcare patients and staff. However, there is a scarcity of information about trauma-informed care in long-term care settings (Department for Levelling Up, Housing, and Communities, 2023) and in the arts and health sphere (Sunderland et al., 2023).

Participatory arts involve professional artists providing collaborative arts programmes for groups of people in community or care settings with the intention of promoting health and wellness (Fraser et al., 2014; Ward et al., 2020). They differ from expressive arts therapies which are a form of psychotherapy delivered by trained registered professionals, where therapeutic goals and the therapeutic relationship are generally of primary importance rather than the creation of the art itself (Fraser et al., 2014). Comparatively, participatory arts are more concerned with the process fostered by the arts programme (Ward et al., 2020) and recognise the intrinsic value of arts activities rather than seeing them as ‘a means to an end’ (Wilson et al., 2016, 2019).

Participatory arts are known to positively impact mental wellbeing, including for older people in care settings (e.g., Fraser et al., 2014; Paolantonio et al., 2020). Research demonstrates that people who have experienced trauma gain wellbeing benefits from engaging with expressive arts therapies, however little research exists exploring the impacts of participatory arts for people who have experienced trauma, despite such work taking place (see Baring Foundation, 2023). The limited research that has been published highlights the potential for participatory arts to support the mental wellbeing of specific groups who have been/are likely to have been exposed to traumatic events, such as veterans (Vest et al., 2025), refugees (Lenette and Sunderland, 2016), and abuse survivors (Rouse et al., 2023). Recent research has also highlighted the wellbeing benefits of participatory arts delivered to school children remotely during school closures associated with the COVID-19 pandemic (Mastrothanasis et al., 2025a, 2025b). The Culture, Health, and Wellbeing Alliance (2023) recently asserted that participatory arts organisations should embed a trauma-informed approach, however there is little research on trauma-informed practice by participatory artists (Sunderland et al., 2023).

Lenette et al. (2016) developed a framework for participatory music to counter the impact of traumatic experiences on asylum seekers’ wellbeing, comprising four intertwined concepts: humanisation, community, resilience, and agency. More recently, based on a review of the literature which combines oppression-related trauma-aware practice with arts activities (including arts therapies), Sunderland et al. (2023) developed principles for anti-oppressive and trauma-aware practice in health and community arts. Some of these principles echo existing trauma-informed frameworks, such as practitioners needing to develop a background awareness of trauma and to generate safe spaces. Additional considerations specific to arts practice and the participant group, included the arts providing verbal and non-verbal opportunities to express and release trauma. Also advocated by Birch (2022), this highlights the need to develop contextually appropriate trauma-informed frameworks of practice in the arts and health field.

In this paper we present a trauma-informed framework for participatory arts practice in the residential care home context, having applied learning from the responsive approach of a UK-based programme (Wilson et al., 2023) that took place throughout the COVID-19 pandemic.

## 2 Materials and methods

### 2.1 ARCH programme context

ARCH drew on Magic Me’s specialist knowledge of bringing arts into care homes and connecting meaningfully with residents. The ethos behind Magic Me and the ARCH programme was to provide meaningful, high quality participatory arts activities that are otherwise inaccessible to residents, with the aim of improving overall quality of life and promoting social justice. The aims of ARCH were to improve relationships and understanding between care home staff and residents; raise expectations in the care sector of what the arts can achieve in care homes and inspire the care homes to integrate the arts into the homes culture; and increase the artists’ understanding, skills and confidence working in the sector.

The participating arts organisations were recruited by Magic Me as Arts Council England National Portfolio Organisations that had experience of using different forms of participatory arts with various groups but had not necessarily worked in care homes previously.

### 2.2 Care home settings

Magic Me engaged in desk research and had conversations with Essex County Council colleagues and other local partners to generate a shortlist of care providers. Magic Me then had initial discussions with those shortlisted providers. There are few care home providers operating at scale in Essex. One care home management company owned 31 homes, across a range of locations—coastal, small town, small city, edge of London—giving the scope and variety Magic Me desired for ARCH. Magic Me directors and the Essex Operations Director of the chosen care home management company then worked together to shortlist homes in Essex with capacity to join the project. Magic Me visited each of the shortlisted homes with the care home management company’s Executive Head of Lifestyle and Innovation, meeting the care home managers, staff and residents. Following discussion, Magic Me and representatives from the care home management company selected four homes and matched them each with an arts partner based on what they had shared about their work and ambitions for ARCH.

The participating care homes varied in size from 40 to 92 beds, offering residential, residential dementia, respite, and end-of-life care. Various activities were already being delivered in the homes, but this was primarily passive engagement with performers/entertainers. ARCH provided a new opportunity to engage with immersive participatory arts through long-term partnerships with arts organisations.

### 2.3 ARCH programme delivery

ARCH took place from 2019 to 2024 over three phases: R&D (allowing time for artists and care homes to get to know each other and for artists to experiment with creative activities in their partnered home), year-long artists’ residencies, and legacy and embedding. The programme also included training for artists throughout. Throughout the programme, ‘cohort days’ were hosted by Magic Me, where participating artists, care home staff, and representatives from the care home management company met together for introductions, discussions and/or training. The original intention was that these would be in-person, but the last two cohort days were held online due to pandemic restrictions.

### 2.4 Research design and data collection

Keen to capture and share learning, Magic Me approached ARU to be their research partner. Both Magic Me and ARU had been part of Creative Journeys in 2017 (Bungay et al., 2019). The research team adopted a collaborative research design, involving Magic Me as research partners in shaping the research aim, data collection process and providing iterative feedback on draft findings. The research aimed to explore how arts and care home staff worked together to successfully deliver and embed participatory arts in care homes. The overall research questions were: how do artists and care home staff collaborate to deliver and embed participatory arts in care homes? What challenges and enablers influence this process? And what constitutes best practice for artists delivering participatory arts activities in care home settings?

The research took a qualitative approach that encouraged collective and individual reflection by the artists, care home staff, the care home management company and Magic Me throughout the phases of the ARCH programme. Qualitative research methods are reported here according to the consolidated criteria for reporting qualitative research (COREQ) (Tong et al., 2007). Details of the research methods during each phase of ARCH are outlined below. The total data corpus comprised 11 observations, five interviews, and 34 focus groups. All authors undertook interviews, focus groups and observations between them. The researchers have established expertise in the arts and health field and previously conducted research in residential care homes (Dadswell et al., 2020; Bungay et al., 2021). All interviews and focus groups were conducted using flexible interview guides and were audio-recorded and transcribed. Ethical approval was granted by the ARU Education and Social Care School Research Ethics Panel.

### 2.5 Participants

The total number of participants involved in the observations, interviews and focus groups over the four-year programme were as follows: 11 artists, two Magic Me directors, four care home managers, one deputy care home manager, six care home lifestyle coordinators (sometimes referred to as activities coordinators), thirteen care home carers, and three representatives from the care home management company.

### 2.6 Cohort day 1

In September 2019 an in-person introductory day brought together people from the participating arts organisations, Magic Me, representatives from the care home management company, and the ARU researchers. A dementia specialist and trainer co-led the day, offering an introduction to working creatively within care homes.

On the cohort day the research team introduced themselves and the research aims. They provided Participant Information Sheets (PISs) and answered questions before obtaining written informed consent. The researchers used the ‘sticky notes’ method to invite arts organisations and the care home management company to respond to four questions: how they felt about the prospect of delivering ARCH, what was seen as exciting, what were the anticipated challenges, and what skills would be needed. A focus group at the end of the day reflected on the day and what was needed going forwards.

### 2.7 Cohort day 2

Each arts organisation spent a day at their partner home (along with Magic Me, representatives from the care home management company and two of the research team) which included: an introduction from the manager, Lifestyle Coordinator, and other key staff; a tour to meet residents/staff; and an introduction to the arts partner’s practice through talks, films, and experiential activities.

The researchers re-introduced their role and approach and provided PISs and obtained written informed consent from those who were not at the first cohort day. The researchers conducted unstructured written observations throughout the day and a focus group at the day’s end, which reflected on the visit and hopes and concerns. Follow-up telephone interviews were conducted with care home managers.

### 2.8 R&D phase

Between November 2019 and March 2020 arts organisations spent time in their partner home, getting to know people, learning about how things worked, and experimenting with creative activities. Their goal was to shape an appropriate, longer residency in the next phase. At the end of the R&D phase online focus groups were conducted with each arts organisation, care home, and Magic Me which reflected on their experiences through the R&D phase.

### 2.9 Cohort day 3

In April 2020 cohort day three was held online due to recent initiation of lockdown in the UK. Understandably care home and care home management company staff were unable to attend. The research team shared the findings so far and asked the arts organisations to reflect on the findings. One of the researchers took notes.

### 2.10 Pausing and restarting

From March 2020, care home staff were fully focused on dealing with the pandemic. Arts partners paused all planned work, cancelled tours, and renegotiated funding agreements. Some arts partner staff were furloughed. From early 2021 Magic Me brokered online meetings for homes and arts partners. Due to the trauma faced by care home staff and residents, Magic Me arranged a session for artists with a consultant and counsellor where artists reflected on how they might respond to the situation in their care home.

As artists and care homes began to reconnect, strict national guidelines on visiting care homes remained in place. Contact was initially via phone/Zoom, though some garden visits/outside work was possible in 2021. Magic Me initiated monthly online meetings for the artists to share experiences, questions and learning as they experimented with remote and then in-person work. The researchers joined the artists’ monthly meetings and took observational notes.

### 2.11 Main residencies

Care homes opened fully to visitors in April 2022, though COVID-19 outbreaks could still mean a temporary closure. Between April 2022 and March 2023 artists delivered workshops, performances, and staff training inside the homes, in outside spaces, and via Zoom, interactive films, and activity pamphlets. They used various mediums including dance/movement, textiles, music, performance, film, virtual reality, digital storytelling, science experiments, and photography. Some created films with residents/staff and held film screenings. In some homes, artists installed soundscapes, sensory rooms, videoscapes, and film studios. Artists left behind creative outputs and digital workshops or activity boxes. This included interactive films, scrapbooks, photobooks, sound installations, and tactile objects.

Online focus groups were conducted pre-residency with each arts organisation, care home (usually with the Lifestyle Coordinator and/or the care home manager, sometimes joined by wider care home staff), care home management company representative, and Magic Me to explore what might have changed since the end of the R&D phase and their expectations for the residencies. After the residencies, online focus groups were conducted with each arts organisation and care home.

### 2.12 Cohort day 4

The final (online) cohort day was held in March 2023 and was attended by the arts organisations, care home staff, Magic Me, and the research team. Three online focus groups were conducted in separate breakout rooms: (1) all arts organisations, (2) all care home staff, and (3) Magic Me. The ‘sticky notes’ method was adapted for the online space to reflect on: what they wish they had known at the start; what had been the key learning points; and what they would take forward into the future.

### 2.13 Legacy phase

The legacy phase took place alongside the residencies and continued until March 2024. A written resource booklet of creative ideas and approaches from the residencies was produced and disseminated to Lifestyle Coordinators across the county.

### 2.14 Data analysis

Each dataset was explored using inductive reflexive thematic analyses (Braun and Clarke, 2006, 2019) to draw out key themes. Analysis involved deep and prolonged immersion in the data, alongside ongoing reflection and thoughtfulness, moving iteratively through phases of familiarisation, initial coding, theme development and review, and the definition and labelling of themes. All authors were actively involved in all stages of the analytic process. Each author individually read and re-read each data set to identify initial codes and then came together to identify patterns of shared meaning which were developed into themes through iterative discussions. These discussions supported reflexive consideration of the data and developing themes, considering how the authors’ positioning and perspectives informed the construction of codes and themes. This process took place initially at the end of the R&D phase for the authors to present interim findings at ‘Cohort day 3′ and then took place again at the end of the residencies phase after ‘Cohort day 4′ whereby authors analysed the further data sets and revisited the R&D phase data set. Whilst analysing the latter data sets, the authors identified themes which related to COVID-19-related trauma and approaches of artists which facilitated trauma recovery. The authors noticed alignment between these approaches and broader principles of trauma-informed care and structured the themes into a trauma-informed framework for participatory arts practice in residential care homes.

The findings presented in this paper draw on the data related to COVID-19-related trauma experienced in the care homes, and the approaches of the artists which facilitated trauma recovery. Illustrative quotes are presented from across the phases, “/” is used to indicate a change in speaker in quotes from focus groups. Our findings that relate to the importance of the artists and care home staff building trusting relationships and working collaboratively to allow for the generation and embedding of sustainable creative practice in the homes, are explored in Dadswell et al. (2020).

## 3 Findings

The first theme considers the context and experiences of COVID-19-related trauma in the care homes. Subsequent themes reflect the approaches that artists used to support trauma recovery in care homes: creating safe and inclusive spaces, training and support for artists, building trusting and collaborative relationships, empowering care home staff and residents, fostering joy, and channelling the power of the participatory arts. We then present our trauma-informed framework for participatory arts practice in residential care homes. Whilst we refer to ‘artists’ and ‘arts organisations’ in the findings, the principles discussed can be applied and adapted for use by other facilitators, if resources do not allow for bringing professional artists into a care home.

### 3.1 COVID-19-related trauma in the care homes

As with many other care homes around the world, the participating homes experienced devastating losses, with high resident and staff turnover, and extreme staffing shortages. Care home staff worked very long hours with long stretches without days off, and senior staff from the care home management company worked shifts in care homes at the height of the pandemic to try to help ease the pressure. Many residents died from COVID-19, and staff described their roles “like [that of] a funeral director”. In one home, half their residents died due to COVID-19 within 2 months.

The care home staff painted a picture of fear, sadness, grief, and guilt through the peak of the pandemic, and beyond. They described the immense pressure they had been under and being unable to process what had happened nor release their emotions. Several staff described feeling incredibly scared at the start of the pandemic due to fear of the unknown, fearing for residents’ lives, their own lives, and the lives of their families. By the time ARCH restarted, there was no sense of security or complacency amongst care home staff as they remained fearful that the situation would worsen again.

…you don’t really tend to think about what has happened…but when we do…it does hit home…it was the most scariest thing, it felt like you were just dealing…with like a wartime but fighting nothing. / Because you couldn’t see anything. / …And all of a sudden what we signed up to do, well they put our lives at risk because at that time no one knew if it was going to kill you or not. (Care home focus group pre-residency)

I don’t even remember the hours I have done some weeks. Seventy hours. /…working like 28 days in a row, 12 hours shifts. / I don’t think we ever had the time to process that…we were emotionally affected really badly… /…there was a night where the ambulance people came and took six people in one night. /…I think coming out, through it all…it is tougher now…people are now realising…we have been damaged by it. (Care home focus group pre-residency)

Staff described having only been able to provide basic care for an extended period, and staff and relatives expressed concerns that residents had become isolated and lacking human touch. Although this was due to the pressure staff were under alongside government-imposed restrictions, the resultant care provided and the isolation of residents left staff feeling guilty, and some relatives angry. Managers had been contacted by relatives concerned that care home staff had become less able to provide compassionate and empathetic care due to the trauma they had faced, and staff felt they needed support to heal from their own trauma to be able to care properly again. We were told that staff morale was very low, as staff felt they were abandoned and “the forgotten people” during the pandemic. Staff were “*desperate*” for support, and for ARCH to start again to restore joy, provide compassion, and space to release and heal from the emotional trauma. However, other care home staff were dismissive of the idea of the artists supporting their own wellbeing and wanted the artists to only focus on the wellbeing of residents, perhaps due to remaining feelings of guilt.

…the relatives have been complaining…that the staff don’t seem to have any empathy any more…there is a slight friction…between the care that’s being received… and what the families perceive is the right amount of care and kindness that their relatives should be receiving… and I think [management company] will want us to…encourage [staff] just to drop their boundaries…which is incredibly difficult. What a hard thing to ask after you’ve been through a pandemic and you have to protect yourself and deal with all this death and sadness, to then drop your boundaries and be open to it again. (Arts organisation focus group pre-residency)

We had an overwhelming conversation with the care home staff – seeing the weight and trauma of everything they’d been through over the last year…the care home staff were so strong in their expression of a need for fun. They also kept saying that “it’s for the residents, don’t worry about us” but we asked, “how do we ‘demand’ that this is something that nourishes them too, as it would be good for everyone.” (Artist at March 2021 meeting)

The artists shifted focus to consider staff wellbeing and recovery from trauma when they returned to working with the homes. At one of the artists keep-in-touch days, artists were asked to write a six-word story about how they were feeling about ARCH starting up again. Eight artists wrote a story, including “moving forward with tentative energized steps”, “deep breath – meeting, touching, being again” and “hopeful, deeply sad, unsure, sure, excited”. One of the artists’ six-word stories was particularly poignant and eloquently encapsulated the group narrative during the meeting:

“*Starting from scratch, trauma to recovery*.” (Artists keep-in-touch day)

### 3.2 Creating safe and inclusive spaces

The artists created spaces which promoted physical safety, particularly to protect staff and residents from COVID-19, as well as considering safe and inclusive working for residents with physical and cognitive impairments. It was important for artists to create spaces, and interactions, that promoted emotional safety and offered opportunities for staff and residents to express and release their emotions.

Artists were aware of staff anxieties about COVID-19 as ARCH restarted and ensured they adhered to the home’s COVID-19 regulations and considered use of online and outside spaces. To reduce risk of transmission, and make staff feel safe the artists worked to gain trust, were sensitive, respectful, and willing to adapt ideas and practices.

…we’ve just been scared introducing this back into the home again. Obviously, we’re so governed by our guidelines…and we thought, “Well, how is this going to be done?”…But they were fantastic. They built an amazing stage [outside], and the residents had a great time…they did a puppet theatre…they were dancing [with the residents] with a bit of string in between them…They were so respectful…Because at any time, if…we thought that wasn’t being met, then we would’ve had to have said, “We really just can’t do that.” (Care home focus group pre-residency)

The artists thought carefully about when and where to set up spaces away from busy communal areas, when to mix into communal spaces, and created activities that could move between spaces and accommodate those confined to their beds, such as making tactile blankets with residents and creating soundscape books with images that could be taken into residents’ rooms.

Some artists repurposed areas in the homes into beaches, jazz clubs or film studios, to create spaces for reflection and relaxation. In one home the artists installed a beach area, with accompanying music, sounds, lights, and images. Care home staff described how it gave time to reflect, relax, and “*deal with*” their emotions.

…with the beach…we’ve got an area on the second floor where they’ve done it all up…it has given the impact to everybody, not just the residents. We’ve now got staff members using the facility to be able to take five minutes out…We deal with a lot of sadness within the home…and I don’t think we ever really deal with it in a way that perhaps we should…So it just takes you to think about things. And actually just having that piece of music and just sitting there, and turning my back on all that’s going on, and just dealing with that emotion. And it’s okay to cry, it’s okay to show emotion…has just been amazing… (Care home focus group post-residency)

To be inclusive, these spaces also needed to accommodate the needs of residents living with dementia:

We’ve got lights, that give off a…half-moon-type…or like a full sun, if you have it bright…So if it was to be somebody that was very agitated, to go to that area, you’ve got the light, you’ve got the music, it will definitely help. (Care home focus group post-residency)

These physical areas also provided space for residents to express and release emotions. In one home the artists transformed an unused room into different scenes and environments for the residents to experience, explore and respond to through movement and music, which they filmed as a lasting memory for the home.

…the power of movement…really came out with one particular resident. It was the power of moving in her way, but moving with us and moving over time…It was really her expressing herself, and in the middle of this improvisation, she stopped and she started crying. She said, “I don’t know what’s happening…”…We said, “We completely do know what’s happening. You are expressing yourself,” and she took a deep breath and she carried on. (Arts organisation focus group post-residency)

The use of a dedicated room also served to build curiosity as staff members would knock on the door, intrigued as to what was happening inside. This helped to gain their interest in advance of when the artists “*took over*” the communal lounge for a “big theatrical moment” where the artists wanted to include as many people as possible, including the kitchen staff.

To promote emotional safety, and allow care home staff and residents to feel safe and comfortable expressing themselves, the artists were welcoming, friendly, fun, calm, compassionate, empathetic, accommodating, patient, intuitive, and responsive. Residents responded to this and wanted to talk with them and engage with the activities.

…the residents respond so well to them…because of their way, their personality…I said if we could bottle that, just hearing them laughing and joking…Even with the masks, their faces just lit up…it is their persona and the residents react to that. They’re kind of drawn to it…they managed to actually coax [new things] out of the residents…in a fun way, it wasn’t intrusive. (Care home focus group post-residency)

### 3.3 Training and support for artists

The artists received training about the trauma care homes had faced, which allowed them to develop their practice. They also received training on the varying needs of residents, the day-to-day lives and routines for residents and staff, general challenges that staff and residents face, as well as specific challenges faced during the pandemic. This raised awareness and created empathy amongst artists, enabling them to work effectively and appropriately with staff and residents.

…it had come across quite strongly in a number of those conversations [between care homes and artists] how awful it had been for the staff. [Artists] were asking about, “…I want to understand that a little bit more or know what I can do to support that…” So, I…found a consultant and counsellor, who…developed a half-day training, which was about grief and trauma in care homes through the pandemic…So, you yourself as an artist will have a response to what has happened and the trauma, as will the residents and the staff that you are working with. So, it was giving the artists a vocabulary to talk about that and to think about how they might need to shift both their communication with the homes, but also the practice and the way that they work with the residents and the staff… (Magic Me interview post-residency)

Within ARCH, there were mechanisms in place to support the wellbeing of the artists, who could experience secondary trauma from working with the homes but had also faced their own personal traumas during the pandemic. This included support from Magic Me and external specialists, as well as peer support between and within the arts organisations. During a meeting in March 2021, the artists expressed the need for regular peer support so Magic Me organised monthly online meetings for the artists to support each other through the return to working with the homes. Alongside this, each arts organisation ensured that there was appropriate supervision and debriefing for artists within their own organisation.

…it can be a space which feels incredibly challenging to be in…I’ve been very much grappling with how we keep artists safe…My two artists that are still working in the space have both expressed challenges around their mental health in relation to the project…just how upsetting it is to hear about what’s happening in the home… (Arts organisation focus group pre-residency)

### 3.4 Building trusting and collaborative relationships

For the participatory arts activities to have meaningful impacts, the artists needed to build trusting and collaborative relationships with the staff and residents particularly as they were often proposing things that had not been done in the home before. Developing trust was vital in allowing for meaningful collaboration, however, it took time. Artists spent time being ‘present’ in the homes, and maintained communication with staff within and between sessions, to build relationships and shared understandings.

…your artistic endeavour is not the most important thing. The important thing, initially, is to get to know those people as people, that home as a home. What happens, what they’re interested in…that layer of building the relationship… (Arts organisation focus group post-residency)

…little by little… [care home staff] noticed…we took the time with individual residents. So that allowed them to go, “We trust these people…as humans. We trust…that what they’re doing is of value.” (Arts organisation focus group post-residency)

To work collaboratively, everyone needed to be open to change, artists did not go in with fixed ideas but involved care home staff from the start creating ideas together. Artists defined care home staff as “*experts*” and valued their knowledge, skills, and experience.

Rather than…“We’re going to come in and show you how we do…” it’s like, “…you are the experts. Can you teach us, as artists, how this care home works day-to-day and how you work?” (Arts organisation focus group post-residency)

Artists took time to come together with the care home staff in the form of check-ins before and after sessions, which allowed staff to inform artists of any changes in the home that might impact their work, reflect together on what worked and incorporate learning into future sessions, and promote mutual feedback and idea-sharing to ensure equality of relationships. However, whilst artists encouraged care home staff to voice their ideas and provide feedback, there were concerns that they may not feel confident to share if something was not working well. Empowering staff and building their confidence was vital.

### 3.5 Empowering care home staff and residents

Care home staff, and residents, needed to feel empowered to collaborate and have a voice throughout ARCH. This was facilitated through building their confidence, valuing their expertise, building on, and celebrating their strengths, and instilling pride and a sense of ownership.

At the artists’ meeting in March 2021, the artists discussed the importance of “…*communicating what we need and what they [care home staff] need and then going through a process of co-creation*” to facilitate staff feeling “*more empowered*.” They reflected that through mutually identifying “what each individual roles are and where the space is for those roles to expand and flex” gives “staff permission that they can be in the space and how they can be in the space”. Additionally, at the October 2021 cohort day, one of the artists reflected that there was great “importance of Voice and Choice for residents” following the powerlessness they had faced during the pandemic.

The artists discovered and built on the skills and strengths of the staff and residents, but to do this they needed to be present. One artist shared how they discovered that one care home staff member was trained in Indian dance, which others in the home were not aware of. The artist noted that “who they are isn’t being brought into that care home” and that through “giving them space to talk about that” their strengths and skills were revealed. At the final cohort day, the artists reflected on how their approach to working with the staff, gave staff permission to be creative, have fun, and move beyond a purely clinical role. This then gave the staff confidence to share ideas and parts of themselves they had previously hidden.

I think this whole thing about inviting them or giving them permission to think beyond what they already know… [artists] presence or the presence of the different things that we've now bought into their space as a kind of invitation to think beyond. And that's what's been really heartening…suddenly, they're flooding with ideas, and ways that they're going to build on the things that that we have started with them…and using all those skills and amazing things that they already have and bringing that into the space where perhaps they maybe didn't feel that they had permission to do so before. (Cohort day 4 arts organisations focus group)

One of the care home staff members also reflected on this:

…we’re not a hospital, we’re a care home, and it’s where people are cared for, in a home environment. And I think that…having the artists in has actually- It’s okay to be like this. It’s okay to dance around and have fun…you're not here just to do clinical work, this is a home. (Care home focus group post-residency)

Artists also expressed the importance of seeing and responding to residents as creatives, and helping care home staff to see the residents in different roles.

…this project for them has brought out the many other sides of the residents that they [care home staff] don’t necessarily see…A project like this allows those people to reinvent themselves and have a different way. [Care home staff] were talking specifically about [female resident] in the film with that solo movement and they were saying actually their day-to-day relationship with [her] is that she’s a very lovely polite quiet woman who likes a gentle chat. Actually, they saw her as a soloist. (Arts organisation focus group pre-residency)

The pride of staff and residents resulting from the programme was clear:

…they all felt proud of this film studio…One of the worlds that we created was what we called ‘Jazz World’. We made a jazz club…hence the smoke alarm things, lights, red cloths, and things. Actually, [care home manager] came and brought her daughter, and [care home staff member] would bring people in. New families were coming to have a look around the home and he proudly brought them and said, “Oh, this is what we do. We’ve got a film project, theatre project happening.” (Arts organisation focus group post-residency)

To say that we’ve done that in a care home and that it’s worked, you know. We shout about it… (Care home focus group post-residency)

The films created by two of the arts organisations celebrated the residents and care home staff. They were made for the residents and care home communities with the focus on their experience of engaging, making, and telling stories that were captured to provide a lasting memory.

[The film] has had an effect from the residents all the way through to management…I don’t think they would have engaged in the same way without the output of the quality of film that has been made, and seeing that…level of art in this space…and it’s about celebrating them. (Arts organisation focus group post-residency)

Film screenings brought the whole care home together to experience the impact and power of the arts which was a particularly special event for residents, described as “one of the best things for them…to see themselves in the film”, but also facilitated staff reflection on their role and relationship with residents.

[Watching the film] made them realise how important their connection…with the residents was…they could see…how much they…were the family to the residents…really seeing both the importance of what they do, and how they can do it more… (Arts organisation focus group post-residency)

### 3.6 Fostering joy

As stated above, the care home staff expressed a need for joy, fun and laughter to be brought into the homes. They later articulated how this was achieved, with the artists lifting their spirits, fostering happiness, and restoring wellbeing.

…we didn't think that we were ever going to be…back to our baseline…because we'd had so much sadness and misery…To hear music and laughter, again, in the home, has just been amazing. It kind of lifted us all. It lifted all our spirits. (Care home focus group pre-residency)

Furthermore, the skills that the artists had passed on to the care home staff, continued to positively impact resident wellbeing beyond the residency.

…when I was using what I’d seen in the [artist’s] sessions…this 98-year-old gentleman… looked at me and he said, “You were put on this earth to make people happy and you really do.” That really touched my heart because the difference that you’re making through this means of communication, that isn’t to do with talking and language and trying to understand everything, is just phenomenal. (Cohort day 4 care homes focus group)

### 3.7 Channelling the power of the participatory arts

Through conversations with artists and care home staff, it became apparent that participatory arts can be powerful in restoring joy, releasing emotions, and aiding recovery from trauma. The artists channelled the power of the arts in their work with the homes, which led to many “special little moments” where the arts generated meaningful impacts.

In the post-residency focus group with one of the homes, they reflected on how ARCH delivered “far more than” the performers and acts they had brought into the home before.

…what I’ve found with this, especially towards the end, was the impact it has had on everybody…For me, the piece of music that was put together was breath-taking…It was art to another level… (Care home focus group post-residency)

The power of the arts was also conveyed by the artists in realising the importance of their work in the care home setting for residents who are apart from their families and coming towards the end of life. This was particularly true for artists who had not worked in care homes before.

I think the ones [artists] that hadn’t had any experience in a care home before were quite profoundly moved by the whole thing…it was just so special to see that reaction of: “This is the power of the arts. I can see it up close and personal. I can feel the impact that it's having.” I think when you’re working on a big show and you’re backstage, you don’t necessarily get the audience’s reaction…but especially for the technical stage manager…for him to be so connected to that power that we all know that arts can have…it was one of the first times that he’d seen it so viscerally… (Arts organisation focus group post-residency)

Reflecting on the power of the arts, care home managers expressed how “there is so much need” for programmes like ARCH and the pandemic made these findings even more pertinent as both artists and care homes talked about how long the residents went without having contact with anyone apart from the care home staff.

I think it is such a special thing to be able to do. And I think one thing that has come from the pandemic is it has shone a light on care homes…because of all the stuff that went on in care homes and how awful it was…last summer…we were outside, the residents were inside but behind the French windows…and it was the first thing they had had through the whole pandemic…the first entertainment or people coming in. And you could see they were so excited just to have different people…something new happening. I think it has made people think about that sort of situation. What it is like to just be stuck somewhere where you need things to come in and make people’s lives better, you know? It is so important. (Arts organisation focus group post-residency)

The tangibility of creative outputs (such as films and photobooks) was also “*really powerful*” in positively impacting wellbeing beyond the life of the programme. As one Lifestyle Coordinator remarked, “you’ve got it forever”.

### 3.8 Trauma-informed framework for participatory arts practice in residential care homes

Our findings presented above have informed our trauma-informed framework for participatory arts practice in residential care homes. Our framework comprises six inter-connected principles: creating safe and inclusive spaces, training and support for artists, building trusting and collaborative relationships, empowering care home staff and residents, fostering joy, and channelling the power of the participatory arts. Above, we have presented examples of how these principles led to meaningful wellbeing impacts, and moving towards recovery from COVID-19-related trauma for care home staff and residents in the four care homes in our research. During the analytic transition from themes to the framework, themes were interrogated in relation to the practical actions, activities and artist/facilitator approaches they reflected, generating cross-cutting insights applicable across contexts. These are summarized in Table 1. As explained earlier, principles relating to artists/arts organisations can be applied and adapted for use by other facilitators, if resources do not allow for bringing professional artists into a care home.

**Table 1:** Framework principles and associated activities/actions.

Principle	Activities/actions for artists/facilitators
1) Creating safe and inclusive spaces	Adhere to the home’s regulations. Use a variety of spaces, and carefully consider which activities are suited to which spaces. Consider use of online and outside spaces Create dedicated spaces in unused rooms/spaces for staff/residents to relax, reflect, create, and express emotion (e.g., create a beach or jazz club area). Consider the needs of residents with dementia when designing the spaces. Consider activities in communal spaces to include a large proportion of residents and staff. Use activities/artworks that can move between spaces to accommodate those who are immobile, e.g., portable soundscapes, photobooks, tactile blanket-making. Be sensitive and respectful regarding staff and resident concerns about virus transmission. Be adaptable and flexible. Be welcoming and friendly. Be calm, patient, compassionate and empathetic. Be responsive and intuitive.
2) Training and support for artists/facilitators	Attend training on the care home context. Attend training on grief and trauma in care homes. Allow time to immerse yourself in the life of the care home. Access peer support, supervision and debriefing from/with other artists/facilitators.
3) Building trusting and collaborative relationships	Spend time getting to know staff and residents and gaining trust. Communicate with care home staff in direct and clear ways. Define care home staff as experts and value their knowledge, skills, and experience. Create time and space for check-ins and debriefs with care home staff. Promote feedback.
4) Empowering care home staff and residents	Build care home staff and residents’ confidence. Value staff and residents’ expertise. Build on and celebrate staff and residents’ skills and strengths (and spend time getting to know their skills and strengths). Encourage, and make space for, sharing of ideas. View and treat staff and residents as artists/creatives. Give staff ‘permission’ to be creative, to have fun, and to move beyond a purely clinical role. Instill pride and sense of ownership over creative outputs. Create outputs (e.g., films) that celebrate staff and residents.
5) Fostering joy	Bring fun and laughter into the home through different art forms and approaches to interactions (alongside creating spaces for emotional expression and release and being sensitive to the trauma they have faced).
6) Channelling the power of the participatory arts	Channel the inherent power of the arts to restore joy, release emotions, and aid recovery from trauma (e.g., through use of appropriate music, dance, film-making). Draw on the power of the arts to communicate in non-verbal ways with residents with dementia. Create lasting tangible creative outputs with staff and residents.

## 4 Discussion

Care home staff and residents faced significant trauma during the COVID-19 pandemic, and trauma-informed approaches have previously been presented as a solution to addressing the collective trauma of the pandemic for healthcare patients and staff (Fenney, 2021). In this paper we argue that trauma-informed approaches can be impactful in facilitating recovery from COVID-19-related trauma amongst care home residents and staff.

In this research, descriptions of care home staff and residents’ experiences during and after the COVID-19 pandemic reflect existing definitions of trauma as resulting from events that are experienced as harmful or life-threatening and that have lasting adverse effects on functioning and wellbeing (Substance Abuse and Mental Health Services Administration [SAMHSA], 2014). Care home staff described fearing for residents’ lives, their own lives, and the lives of their families. They had lost any sense of security or control and felt unable to safely express their intense emotions. A trauma-informed approach to care is a way of working which seeks to remove the fear, lack of choice and loss of control caused by trauma but creating inclusive, safe services that provide opportunities to rebuild empowerment and control. We found that artists in the ARCH programme who delivered participatory arts activities in four care homes during and after the pandemic created safe and inclusive spaces, provided training and support for artists, built trusting and collaborative relationships, empowered care home staff and residents, fostered joy, and channelled the power of the participatory arts – aligning with the principles of trauma-informed care, and tailored to the specific needs within care homes.

Whilst there is some crossover between our framework and existing trauma-informed frameworks, we found additional considerations specific to arts practice, as well as to the care home context. This supports Birch’s (2022) advocation of the need to develop contextually appropriate trauma-informed frameworks of practice in the arts and health field. Our framework also aligns with, and builds upon, existing frameworks on arts practice (Lenette et al., 2016; Culture, Health, and Wellbeing Alliance, 2023; Sunderland et al., 2023), with additional considerations specific to the care home context. Magruder et al. (2017) previously asserted the need to adopt a public health approach to trauma and that public health trauma advocates may need to form strategic alliances with “*unlikely partners*.” We argue that participatory arts organisations and artists are one such ‘unlikely’ partner.

It is important to recognise that we did not capture the voice of care home residents, as the initial aim of the research was to explore how arts and care home staff worked together to successfully deliver and embed creative arts in care homes. This therefore means that we can only comment on artists’ and care home staff members’ perspectives of the impact of the participatory arts programme on the residents, rather than the perspectives of the residents themselves. Although qualitative research does not aim for generalisability, it is important to recognise that this research took place in four homes based in one region of the UK which may limit transferability of findings. It is also important to acknowledge that some people have critiqued trauma-informed approaches as lacking empirical support and comprising generic principles of good care that are not specific to trauma (e.g., Smith and Monteux, 2023). We recognise that research exploring the application of trauma-informed approaches to care home contexts, as well as to participatory arts practice, is limited. We acknowledge that our framework is based on a responsive approach in only four care homes within one county of England. However, we anticipate, and welcome, further development and evolution of our proposed framework as work in this field grows. Regarding the latter criticism, we agree that there is some crossover between principles of trauma-informed care and wider principles of good care; such as advocating for appropriate training and support, needing to treat people with dignity, respect, and compassion, having empathy, partnership-working, providing inclusive services, using each person’s strengths, allowing time for building relationships, responding flexibly, and listening to feedback (National Institute for Health and Care Excellence [NICE], 2018; Department for Health and Social Care, 2023). However, our framework goes beyond such wider principles of care as we provide principles, and practical examples, that are specific to addressing trauma in the care home context, that are relevant to supporting care home staff as well as residents, and the specific use of participatory arts practice in this context. For example, whilst NICE guidance (2018) mentions considerations for space and environment in care homes, there is no mention of interpersonal safety or creating space for releasing and expressing emotions, nor is there consideration of care home staff wellbeing needs.

In conclusion, we have proposed a trauma-informed framework for participatory arts practice in residential care homes, which is of particular importance considering the trauma that care home staff and residents faced during the COVID-19 pandemic. As care homes continue to recover from the emotional impacts of the pandemic, and continue to face various virus outbreaks and staffing challenges, the participatory arts provide the opportunity to restore joy and promote wellbeing for care home staff and residents.

## Data Availability

The raw data supporting the conclusions of this article will be made available by the authors, without undue reservation.
